# A Multi-Host Approach to Quantitatively Assess the Role of Dogs as Sentinels for Rift Valley Fever Virus (RVFV) Surveillance in Madagascar

**DOI:** 10.3390/v17111461

**Published:** 2025-10-31

**Authors:** Herilantonirina Solotiana Ramaroson, Andres Garchitorena, Vincent Lacoste, Soa Fy Andriamandimby, Matthieu Schoenhals, Jonathan Bastard, Katerina Albrechtova, Laure J. G. Chevalier, Domoina Rakotomanana, Patrick de Valois Rasamoel, Modestine Raliniaina, Heritiana Fanomezantsoa Andriamahefa, Mamitiana Aimé Andriamananjara, Lova Tsikiniaina Rasoloharimanana, Solohery Lalaina Razafimahatratra, Claude Arsène Ratsimbasoa, Benoit Durand, Véronique Chevalier

**Affiliations:** 1 Unité Mixte de Recherche (UMR) Animal, Santé, Territoires, Risques et Écosystèmes (ASTRE), Centre de coopération Internationale en Recherche Agronomique pour le Développement (CIRAD), 34398 Montpellier, France; 2 Épidémiologie des Maladies Infectieuses Multi-hôtes (EPIMIM), Laboratoire de Santé Animale, Anses, Ecole Nationale Vétérinaire d’Alfort, 94700 Maisons-Alfort, Francebenoit.durand@anses.fr (B.D.); 3 Unité d’Epidémiologie et de Recherche Clinique, Institut Pasteur de Madagascar, Antananarivo 101, Madagascar; 4 Département de Recherches Zootechniques, Vétérinaires et Piscicoles, Centre National de la Recherche Appliquée au Développement Rural (FOFIFA), Antananarivo 101, Madagascar; 5 UMR ASTRE, CIRAD, Antananarivo 101, Madagascar; 6 Maladies Infectieuses et Vecteurs, Écologie, Génétique, Évolution et Contrôle (MIVEGEC), University of Montpellier, Centre National de Recherche Scientifique (CNRS), Institut de Recherche pour le Développement (IRD), 34000 Montpellier, France; 7 National Government Organization (NGO) Pivot, Ranomafana, Ifanadiana 312, Madagascar; 8 Unité de Virologie, Institut Pasteur de Madagascar, Antananarivo 101, Madagascar; vlacoste@pasteur.mg (V.L.);; 9 Département de Virologie, Institut Pasteur, 75724 Paris, France; 10 Unité d’Immunologie des Maladies Infectieuses, Institut Pasteur de Madagascar, Antananarivo 101, Madagascar; 11 Department of Immunology, Institut Pasteur, 75015 Paris, France; 12 Institut National de la Santé et de la Recherche Médicale (INSERM), Institut Pierre Louis d’Épidémiologie et de Santé Publique (IPLESP), Sorbonne Université, 93430 Paris, France; 13 ASTRE, CIRAD, Institut National de Recherche pour l’Agriculture, l’Alimentation et l’Environnement (INRAE), Université de Montpellier, 34000 Montpellier, France; 14 Direction des Services Vétérinaires, Antananarivo 101, Madagascar; 15 Centre National d’Application de Recherches Pharmaceutiques, Antananarivo 101, Madagascar

**Keywords:** rift valley fever, Madagascar, serology, force of infection, dogs, humans, cattle

## Abstract

Sentinel animals may play a key role in the surveillance of arbovirus circulation, particularly in developing countries. This study aimed to assess the relevance of using dogs as sentinel animals for Rift Valley fever virus (RVFV) surveillance in Madagascar. Serological surveys were conducted on 513 dogs and 135 cattle in the Ifanadiana district, southeastern Madagascar. In addition, 486 human dry blood samples available from the same area were used. Antibodies against RVFV were detected in 23 of 513 dogs, in 86 of 486 humans, and in 33 of 135 cattle. Serocatalytic models fitted to age-stratified serological data were developed to estimate the RVFV force of infection (FOI) under several hypotheses, ranging from no relationship to proportional RVFV FOIs between humans, cattle, and dogs. The best supported model indicated that RVFV FOI in humans and cattle was proportional to RVFV FOI in dogs. Proportionality parameters were estimated at 2.6 (95% credible interval: [1.4–5.1]) for humans and 3.5 (95% credible interval: [1.3–6.4]) for cattle. Our findings suggested that dog blood samples could be used to identify RVFV circulation in RVF endemic areas and infer the exposure of humans and cattle in these areas in Madagascar.

## 1. Introduction

Madagascar is one of the poorest countries in the world, with 75% of the population living on less than 2 USD per day in 2022, according to the World Bank [1]. Resources for human and animal health are limited, making the population particularly vulnerable to epidemics [2]. It is therefore essential to prioritize the geographical areas where public health measures need to be implemented. Mosquito-borne viruses have a major impact on public health in low-income countries such as Madagascar, and the surveillance of the diseases they cause is usually targeted on vertebrate hosts. Because serological surveys are often difficult to carry out in human populations for both ethical and logistical reasons, the use of sentinel animals can be a cost-effective alternative for monitoring arboviruses [3]. Sentinel animals can be used as early warning of pathogen presence, especially when they are exposed prior to humans or respond to the pathogen more rapidly than humans. They can also be used retrospectively to trace the history of a pathogen’s spread over time and space [4]. An ideal sentinel animal species should be susceptible to infection, survive it, and develop a sufficient and detectable immune response. Moreover, it should not be able to transmit infection to handlers, nor develop a level of viremia that could infect vectors [5]. According to Bowser et al., domestic dogs (*Canis lupus familiaris*) have these characteristics for many infectious agents of public health importance [6], including arboviruses. Several studies have shown dog exposure to flaviviruses, such as West Nile virus (WNV) [7,8], dengue virus (DENV) [9], and Japanese encephalitis virus (JEV) [10].

RVF is a zoonotic disease caused by an arbovirus belonging to the *Phlebovirus* genus of the *Phenuiviridae* family, within the *Bunyavirales* order [11]. Outbreaks are characterized by high mortality rates in young ruminants and abortions in adults [12,13]. In humans, symptoms range from self-limiting febrile illness to hepatic and hemorrhagic syndromes, which can lead to death [14]. RVFV was first isolated in Madagascar from mosquitoes in 1979, without any previous record of human or animal cases [15]. Major outbreaks (epidemics and epizootics) occurred in 1990–1991 on the east coast and in the central highlands, in 2008–2009 on the north and south coasts and in the central highlands, and in 2021 in the southeastern part of the country [16,17,18,19]. In Madagascar, dogs are present in most communities. Apart from their role as pets, they can serve as guardians and/or be used for hunting. Up to 90% of middle-class households own at least one dog in urban areas (such as Antananarivo) [20], while the average number of dogs per household is higher in rural areas (e.g., 1.55 in Ranomafana and 3.23 in Andasibe) [21]. The vast majority of rural dogs roam freely and share the same environment with humans and are therefore potentially exposed to the same pathogens [22]. Two studies have reported RVFV seropositivity in dogs [23,24]. Although these results demonstrate that dogs are exposed to this arbovirus in the natural environments they share with humans, none of these studies have quantitatively analyzed the relationship between the exposure of dogs and humans in a given area.

The aim of this study was to examine the potential role of dogs as sentinels for RVFV in Madagascar by quantitatively analyzing the relationship between the exposure of dogs, cattle and humans to RVFV. To achieve this goal, we used available human sera and collected blood samples from dog and cattle populations in the same area of south-east Madagascar. These samples were serologically analyzed for RVFV antibodies using a commercial competition enzyme-linked immunosorbent assay (cELISA) for dogs and cattle, and a multiplex assay based on Luminex technology for humans. We then used serocatalytic models to test for a relationship between the RVFV Force of Infection (FOI) of dogs and that of humans and cattle, and to quantitatively evaluate the parameters of this relationship.

## 2. Materials and Methods

### 2.1. Study Site and Population

The region, district, commune and fokontany are Madagascar’s first, second, third and fourth (smallest) administrative units, respectively. The study was conducted in 5 communes of the district of Ifanadiana, a rural district in the Vatovavy region in south-eastern Madagascar located 115 km from Mananjary. The Ifanadiana district covers an area of 3970 km^2^, comprises 15 communes and 195 fokontany and is characterized by deep valleys and a mountainous landscape with an altitude gradient of 100 to 1000 m from East to West [25,26]. The landscape features a protected area of dense rainforest in the western part (Ranomafana national park), alongside large agricultural fields and steppe areas. Recorded total rainfall in the district is 1900 mm on average per year, and temperatures vary between 15 °C and 32 °C depending on altitude and season [27]. With an estimated population of 183,000 inhabitants in 2020, the district’s main agricultural activity is rice production [28].

**Figure 1:**
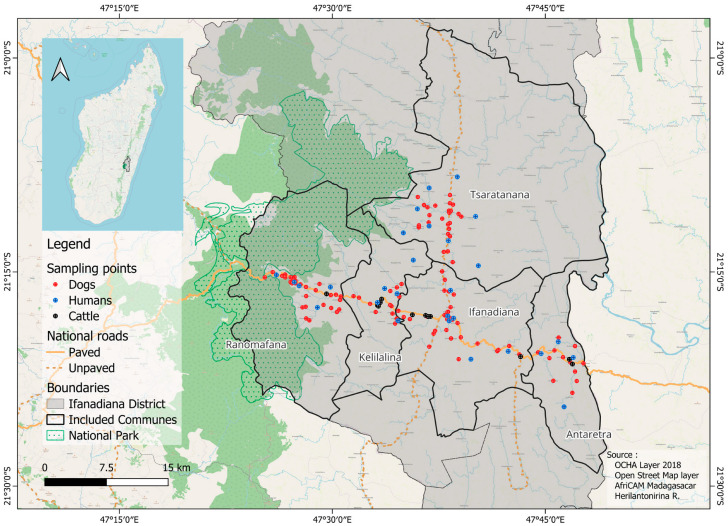
Location of human, dog and cattle sampling points in the Ifanadiana district, Madagascar.

Three host species were considered for this study: humans, dogs and cattle. The selection of the 5 communes was firstly driven by the availability of human samples and associated survey data from an existing longitudinal cohort study (“IHOPE”) [29] described below, and secondly, by the feasibility of sampling dogs and cattle with the help of local veterinarians and local community health workers in the remote areas. The selection of these communes finally took into account their accessibility. These communes were Ranomafana, Kelilalina, Ifanadiana and Antaretra, crossed from west to east by National Road 25, and Tsaratanana which is crossed from south to north by the main unpaved road of the district. Included communes with sampling points are shown in Figure 1.

### 2.2. Serological Data

To ensure that the sampled humans, dogs and cattle had been exposed to RVFV during equivalent periods of time, we targeted children and animals born after 2010, and at least of 3 months old to avoid potential interference from maternal immunity. Data are shown in Table S1.

#### 2.2.1. Dog Data

A cross-sectional survey was carried out in dogs from July to September 2023. To determine the sample size, we assumed that RVFV seroprevalence is lower in dogs than in cattle, in which it had been estimated at 19.3% in animals aged 1 to 12 years [30]. Based on an expected prevalence of 10%, we used Cochran’s formula to set the number of animals to be sampled at 35 dogs per year of birth (for a relative precision of 100% and assuming perfect sensitivity and specificity of the serological test).

We carried out door-to-door visits and identified households owning at least one dog with the support of community health workers and administrative officers (fokontany chiefs). Only fokontany located withing a maximum walking distance of 5 km from the main road were considered accessible and included in the survey. Blood samples were collected from dogs belonging to the surveyed households by teams of trained veterinarians. All dogs living in the area since birth or adopted before the age of three months were eligible and included when possible. Samples were taken by venipuncture of cephalic vein into a tube without anti-coagulant, stored and transported at 4 to 8 °C in a cool box, centrifuged in the field and with the resulting sera stored at 4 °C, then frozen at −80 °C within 3 days at most until laboratory analysis. A free deworming treatment was given to each dog sampled. Age, sex, household location, daily routine as well as dog feeding habits were collected using a structured questionnaire.

#### 2.2.2. Cattle Data

A cross-sectional survey was carried out in cattle in March 2024. Based on an expected prevalence of 19.3% [30], the number of animals to be sampled was 15 cattle per year of birth (for a relative precision of 100% and assuming perfect sensitivity and specificity of the serological test). Due to the rainy season, Tsaratanana commune was inaccessible and excluded from livestock sampling. The remaining four communes were surveyed, focusing on areas where dogs had been sampled in 2023. Only cattle born in the surveyed herd or reported as originating from the survey area were considered for analysis in this study. Farmers were encouraged to present their herd at sampling points designated by local veterinarians for each commune. Some additional herds were identified by snowball sampling. Blood samples were collected by qualified veterinarians by venipuncture of the jugular vein, into a tube without anti-coagulant. Samples were transported at 4 to 8 °C, centrifuged and sera stored at −80 °C. All available cattle were sampled with the owner’s consent. Cattle deworming agents were given to each participating owner. Age and sex of each sampled animal were recorded.

#### 2.2.3. Human Data

Human dry blood spot (DBS) samples from the 2021 collection of the Ifanadiana Health Outcomes and Prosperity longitudinal Evaluation (IHOPE) cohort were used for this study [29]. The IHOPE cohort was established in 2014 to obtain demographic, health and socio-economic information from a representative sample of 1600 households in the Ifanadiana district. The Madagascar National Institute of Statistics (INSTAT) was responsible for data collection, survey coordination, training and oversight. Eighty clusters were randomly selected from mapped areas during the 2009 census [31], and twenty households were randomly selected from each cluster. The 2021 wave of data collection (22 April to 20 June) included, for consenting individuals of all ages, a DBS obtained by finger prick using a single-use lancet needle by trained nurses, with 1 to 5 DBS collected on Whatman 903 Protein Saver Card (WHA10531018, Sigma-Aldrich, Saint-Quentin-Fallavier, Isère, France) filter papers for each individual. All participants (≥15 years) provided oral informed consent for the in-person interview and written informed consent for biological sample collection. Parents or guardians provided written consent for biological sample collection from children ≤ 15 years of age, and children 7–14 years provided written assent separately. In our study, only data from individuals living within a radius of less than 5 km from the nearest dog sampling location were included. All data used in this study were de-identified to protect participant confidentiality. There was no target sample size for human samples, as final sample size depended on the available human data which fulfilled these conditions.

### 2.3. Serological Analyses

#### 2.3.1. Dog and Cattle Sera

Dog and cattle sera were tested using a commercially available competitive Enzyme-Linked Immunosorbent Assay (cELISA) kit (ID Screen^®^ Rift Valley Fever Competition Multi-species, IDvet, Grabels, France). The individual serological status was defined in accordance with the manufacturer’s protocol. In brief, the competition percentage (*S*/*N*%) for each sample was calculated using the following formula in Equation (1):

(1) $$S/N\%=(\frac{DO_{sample}}{DO_{negative\;control}})\times 100$$

A sample was considered positive if *S*/*N*% ≤ 40%, negative if *S*/*N*% > 50%, and doubtful if *S*/*N*% was between these two values.

#### 2.3.2. Human Dry Blood Spots

Antibody detection against the RVFV Nucleoprotein (NP) was performed using Luminex xMAP technology, a microsphere-based immunoassay (MIA, Luminex^®^). Human DBS samples were processed by cutting 3 mm punches, which were eluted overnight at 4 °C in phosphate-buffered saline (PBS-E404, Van Waters & Rogers, Radnor, PA, USA)-Tween 0.5% (P1379, Sigma-Aldrich, St. Louis, MO, USA) under gentle shaking. The eluates were then transferred to 1.5 mL tubes and stored at −20 °C. DBS eluates were dispensed into designated wells and incubated with MAGPLEX COOH-microsphere beads (MC10012-21, Luminex Corporation, Austin, TX, USA) coated with a recombinant RVFV NP expressed in mammalian cells (REC31640-100, The Native Antigen, Kidlington, UK) for 45 min. The beads were subsequently placed on a magnetic plate for 60 s and washed with an assay buffer (PBS containing 0.05% BSA, 0.1% Tween, pH 7.4). F (ab’)2-Goat anti-Human IgG Fc Secondary Antibody, PE (H10104, Thermo Fisher Scientific, Waltham, MA, USA) was added to form antigen–antibody–conjugate complexes. Mouse anti-RVFV nucleoprotein (MAB12334, The Native Antigen, Kidlington, UK) was used for test validation. Following a second wash to remove unbound conjugate, fluorescence intensity was measured using a Magpix instrument (MAGPX12234702, Luminex Corporation, Austin, TX, USA). The fluorescence signal, expressed as median fluorescence intensity (MFI), was quantified and was proportional to the amount of specific IgG antibodies in the sample. To account for variability in blood spot volumes across samples, MFI was normalized to total protein content, determined using a Bradford protein assay [32].

In the absence of a well-characterized set of negative and/or positive reference samples, a modeling approach was employed to classify samples as belonging to positive or negative serological status. A finite mixture model was used to identify clusters within the normalized MFI results across the analyzed population [33]. This approach allowed for the classification of individuals as either low or highly reactive. The model outputs included the number of clusters and the distribution of individuals per cluster, based on the probability of belonging to a cluster. A hierarchical clustering model was then applied to consolidate the identified clusters into two primary groups, that were defined as seropositive (highly reactive) and seronegative (low reactive).

MIA allowed us to detect anti-RVFV IgG antibodies directed against the RVFV NP, while cELISA detected IgG and IgM antibody responses without differentiation. We assumed a perfect specificity (100%) of these tests in each species [34,35,36]. All serological assays were performed at the Institut Pasteur de Madagascar (IPM).

### 2.4. Serocatalytic Models

Serocatalytic models were implemented to reconstruct the RVFV FOI exerted on individuals of the three studied species, defined as the rate of a susceptible individual becoming infected over time [37]. Assuming that the RVFV antibody response is life-long in the dogs, cattle, and humans of our sample (i.e., children aged less than 10 years) [38,39,40,41], the RVFV FOI λ can be estimated by fitting simple serocatalytic models to serological data of individuals of known age. According to this modeling framework, the serological status $Sero_{s,bi}$ of an individual of species *s* with known birth year *bi* follows a Bernoulli distribution, which is shown in Equation (2):

(2) $$Sero_{s,bi}\;\sim \;Bernoulli(p_{s,bi}\;{Se}_{s})$$

where ${Se}_{s}$ is the sensitivity of the serological test used in species *s*, and $p_{s,bi}$ the probability that an individual of species *s*, born in year *bi*, has been exposed to RVFV prior to time $t_{s}$, the year of sampling in species *s*.

We neglected the probability of seroreversion, and *p_s,bi_* was expressed as shown in Equation (3) [42]:

(3) $$p_{s,bi}=1-\exp\left(-\int_{t=bi}^{t_{s}}\lambda_{s}\left(t\right)dt\;\;\right)$$

where $\lambda_{s}(t)$ is the force of infection exerted on individual s at time (year) t.

### 2.5. Hypotheses

Assuming no spatial variation in RVFV FOI within the study area, we tested three hypotheses regarding the relationship between RVFV FOI exerted on dogs (*s = d*) on each year t, $\lambda_{d}(t)$, and on each of the two other species s, $\lambda_{s}(t)$. The null hypothesis (H0) assumed no relationship between $\lambda_{d}(t)$ and $\lambda_{s}(t)$. The first alternative hypothesis (H1) assumed that the RVFV FOI exerted on a given species *s* (cattle or humans) was proportional to the RVFV FOI exerted on dogs, as shown in Equation (4):

(4) $$\lambda_{s}\left(t\right)=\beta_{s}\lambda_{d}\left(t\right)$$

where $\beta_{s}$ was the proportionality parameter for species *s*. For humans (s = *h*), $\beta_{s}=\beta_{h}$; for cattle (bovine, *s = b*), $\beta_{s}=\beta_{b}$. In the second alternative hypothesis (H2), we took into account the fact that dogs may be contaminated either by mosquito bites or by consuming infectious abortion products from cattle (fetus, placenta). Humans can also be infected through direct contact with tissues and fluids of infected animals (especially slaughterhouse staff and butchers as well as veterinary field officers). However, given that we only used human samples from children under the age of 10 (born after 2010 and sampled in 2021), human exposure was assumed to be vector-borne only. In cattle, although direct transmission is possible, vectorial transmission remains the most important route described in the literature. Therefore, in H2, we assumed that the RVFV FOI in dogs was composed of a vectorial fraction $\lambda_{d.v}\left(t\right)\;$ and a direct transmission fraction $\lambda_{d.c}\left(t\right)$ in order to account for the two transmission routes of RVFV. Moreover, the RVFV FOI of a given species *s* (cattle or humans) was assumed to be proportional to the vectorial fraction of RVFV FOI in dogs according to Equation (5):

(5) $$\left\{\begin{array}{c} \lambda_{d}\left(t\right)=\;\lambda_{d.v}\left(t\right)+\;\lambda_{d.c}\left(t\right)\\ .\\ \lambda_{s}\left(t\right)=\;\beta_{s}\lambda_{d.v}\left(t\right)\;\;\;\;\;\;\;\;\;\;\;\;\;\; \end{array}\right..$$

To control for between-years RVFV FOI variations, time was divided into 9 intervals: first, because of the low number of individuals exposed during these years, the period from 2011 to 2016 (with RVFV FOI assumed constant across these years), and then yearly periods from 2017 to 2024 were examined. As the RVFV FOI exerted in cattle in 2024 cannot be calculated from the RVFV FOI exerted in dogs in H1 and H2 (dog sample collection ended in 2023 while bovine sample collection extended until 2024), it was estimated independently.

The sensitivity of the serological tests and the RVFV FOI were hardly identifiable in our models (since sensitivity and exposure probability appeared multiplicatively in Equation (2)). To allow parameter estimation, we thus made the simplifying assumption of a perfect sensitivity in each species.

### 2.6. Model Fitting and Comparison

Combining the three hypotheses (H0, H1 and H2) for the dog–human and dog–cattle RVFV FOI relationships resulted in nine serocatalytic models. According to Equation (2), a binomial likelihood function was formulated to link each model to the data (doubtful was considered as negative) and to estimate the model parameters. Posterior distributions of the parameters were obtained using Bayesian Markov Chain Monte Carlo (MCMC) sampling with RStan plugin (Version 2.32.6) implemented in R (Version 4.4.1). Assuming flat priors for all parameters, 11,000 iterations were performed with 1000 for initial burn-in iterations and a thinning of 5, using 4 MCMC chains, to obtain the median and 95% Bayesian credible intervals (95% BCI) of the parameters. The convergence and efficiency of the sampling process were assessed using the diagnostic metrics Rhat (Potential scale reduction factor) and NEFF (Effective sample size) ratio (Table S2). Autocorrelation was checked graphically with autocorrelation function (ACF, Figure S1). Models were compared using the deviance information criterion (DIC), a measure of model fit. The best model was the one with the lowest DIC. The most parsimonious model (with the lowest number of parameters) was preferred when models had similar DIC values (i.e., when the DIC difference was below 10 [43].

## 3. Results

Overall, 1134 individual samples were considered for the study, including 513 from dogs, 486 from humans and 135 from cattle. Sampling locations are shown in Figure 1, with 113 villages for dogs, 28 household clusters for humans and 14 sampling points for cattle.

The overall RVFV seropositivity rates were 4.5% (95% CI [2.9–6.7]), 17.7% (95% CI [14.4–21.4]), and 24.4% (95% CI [17.5–32.6]) in dogs, humans, and cattle, respectively. The distribution of raw serological results (individual S/N% and MFI values) from cELISA and MIA are, respectively, illustrated in supplementary Figure S2. Serological results by year of birth are presented in Table 1. Maximum seroprevalence of RVFV by species was observed in animals born in 2019 for dogs, 2018 for cattle, and in children born in 2021 (although the sera of only eight children were available for this birth year).

**Figure 2:**
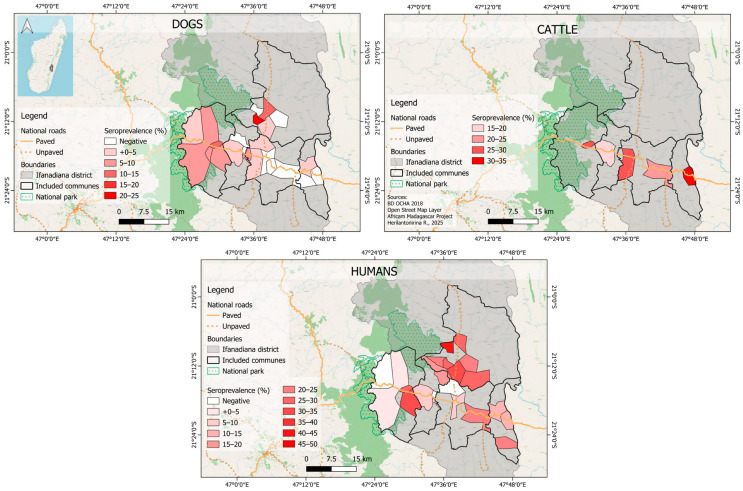
Spatial variations in Rift Valley fever virus seroprevalence in dogs (2023), humans (2021) and cattle (2024) in Ifanadiana district, Madagascar.

For dogs, the RVFV seroprevalence by commune varied between 2.1% (95% CI: [0.4–5.8], Ifanadiana) and 10.9% (95% CI: [4.5–21.6], Tsaratanana). For cattle, it varied between 16.2% (95% CI: [6.2–32.0], Kelilalina) and 33.3% (95% CI: [18.0–51.8], Antaretra). For humans, it varied between 9.2% (95% CI: [4.1–17.3], Kelilalina) and 16.1% (95% CI: [9.3–25.2], Antaretra). Spatial RVFV seroprevalence is illustrated in Figure 2 and presented with more details in Table S3. No consistent spatial RVFV seroprevalence trend was observed across the communes where all three species were sampled.

We fitted nine serocatalytic models to the serological data, for which DIC values and number of estimated parameters are provided in Table 2. The RVFV FOI in humans (respectively, the RVFV FOI in cattle) is modeled as proportional to the RVFV FOI in dogs under hypothesis H1, to its vector-borne fraction only under hypothesis H2, or unrelated under null hypothesis H0.

**Figure 3:**
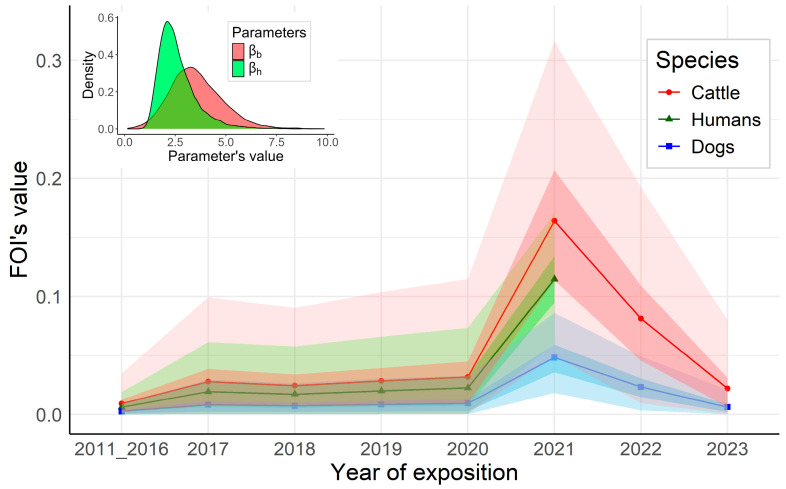
Estimates of the annual RVFV FOI in dogs, humans, and cattle from serological surveys of 2023, 2021, and 2024, respectively, according to the best fitting serocatalytic model assuming RVFV FOI in dogs proportional to RVFV FOI in humans and cattle, Ifanadiana district, Madagascar.

Models 3 and 4 had the lowest DIC values (with no more than 10 points difference between them). Since model 4 was more parsimonious than model 3 (11 estimated parameters for model 4 against 16 for model 3), we selected model 4, in which the RVFV FOIs in humans and cattle were both proportional to the RVF FOIs in dogs. In dogs, before 2021, the estimated median of the RVFV FOI ranged between 0.003 (95% CI: [7 × 10^−5^–0.009]) and 0.009 (95% CI: [2.5 × 10^−4^–0.033]). We observed a significant increase in the RVFV FOI in dogs in 2021, with a median value of 0.048 (95% CI: [0.018–0.086]), followed by a decrease in 2022 (0.023, 95% CI: [0.004–0.049]) and 2023 (0.006, 95% CI: [1.8 × 10^−4^–0.021]). By construction, RVFV FOIs calculated for humans (until 2021, year of sampling) and cattle (until 2023) followed the same trends as the RVFV FOI in dogs, with $\beta_{h}$ and $\beta_{b}$ proportionality parameters estimated for human and cattle at 2.6 (95% credible intervals (CI): [1.4–5.1]) and 3.5 (95% CI: [1.2–6.4]), respectively. Estimates of the annual RVFV FOI are presented in Figure 3.

Model 4 is the best fitting serocatalytic model according to the DIC value and number of estimated parameters, with the RVFV FOI in cattle and human being proportional to the RVFV FOI in dogs.

**Figure 4:**
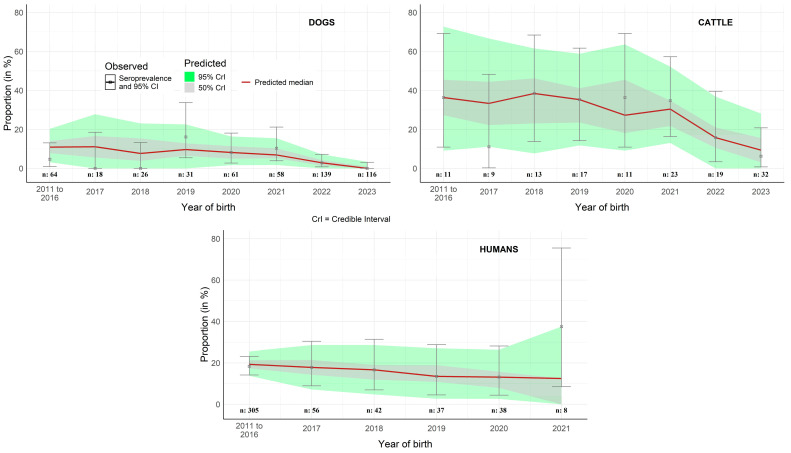
Predicted versus observed seroprevalence of RVFV in dogs, humans and cattle in Ifanadiana district, Madagascar, according to the best fitting serocatalytic model. Gray and green colors represent the 50% and 95% Bayesian credible intervals of predicted RVFV seroprevalence values, respectively, while red lines represent the median values. Points represent the observed RVFV seroprevalence and error bars the corresponding 95% confidence interval. The number of samples per year of birth is indicated by “*n*” values.

The predicted values of RVFV seroprevalence fitted adequately the observed data: whatever the year of birth, the observed RVFV seroprevalence was within the credible intervals of the predicted value as shown in Figure 4.

## 4. Discussion

We estimated the RVFV FOI in dogs, humans, and cattle, using serocatalytic models applied to age-structured serological data [42], and analyzed the quantitative relationship between the RVFV FOI exerted on dogs and the RVFV FOIs exerted on cattle and humans over time. The model that most accurately described the observed serological results (on the basis of the DIC) linked the RVFV FOIs in dogs to RVFV FOIs in cattle and humans by two linear relationships, with proportionality coefficients estimated at 3.5 and 2.6, respectively. This model identified a RVFV FOI peak exerted on the 3 species in 2021, when the last RVF outbreak occurred in the Mananjary district located 115 km from our study site in the south-east of Madagascar. This finding suggested that there was a significant RVFV circulation during this outbreak in the Ifanadiana district, although no clinical cases were reported either in neither cattle nor humans.

To our knowledge, this study reports the first serological detection of RVFV in dogs in Madagascar and is the first attempt to estimate the FOI of RVFV in dogs and its quantitative relationship with RVFV FOIs in humans and other exposed species. The RVFV seroprevalence we observed in Malagasy dogs was comparable to the 3.8% (1/26) observed in dogs sampled in 2018–2019 in a forest area in Gabon [23]. In Egypt, one out of four sampled dogs were reported as seropositive, during the 1977–1978 RVF outbreak [24]. Among the three species here studied, the highest RVFV seroprevalence was observed in cattle (24.4% [17.5–32.6]), followed by the one observed in humans (17.7% [14.4–21.9]). Similar differences in RVFV seroprevalence between cattle and humans were reported in Madagascar during the 2021 RVF outbreak [19], and during the inter-epidemic/epizootic period after the 2008 outbreak [44,45]. These differences between species were consistent with the RVFV epidemiology [46], given that ruminants are considered to be the main reservoir and amplifying hosts of the virus [47,48], while other species such as humans and dogs are considered as accidental and dead-end hosts [24,49]. Moreover, these differences may be due to several factors including body surface area, host attractiveness, and mosquito feeding behavior in the area of concern. Indeed, cattle present a larger body mass and surface area, which increases the amount of CO_2_ emitted and the attractiveness of mosquitoes compared to humans and dogs. While humans are preferred to dogs by some mosquito species such as *Aedes albopictus* and *Ae. aegypti* [50,51], the opposite is true for other mosquito species such as *Culex quinquefasciatus* [52].

We assumed 100% specificity in our serological tests. Indeed, according to a ring trial performed by Kortekaas et al., the cELISA kit (IDVet), which appears to be widely used for RVFV surveillance programs and among the recommended kit for RVFV serological diagnosis [34], demonstrated high specificity in ruminants (100%) [53]. In addition, to our knowledge, the only phlebovirus whose circulation has been reported in Madagascar is RVFV. Our hypothesis of 100% sensitivity of serological tests was a simplifying assumption made to allow parameter estimation. However, it was supported by the fact that the cELISA (IDVet), which is a multi-species kit, has shown high sensitivity in ruminants (ranging from 91 to 100% with mean value of 97.2%) according to Kortekaas et al. [53]. Regarding the MIA, the lack of positive and negative human controls unfortunately limited us to assess the real sensitivity and specificity of the tests performed. However, RVFV NP used as the antigen in the MIA is highly immunogenic [54], and should allow the detection of RVFV antibodies with ease if present. Lower sensitivities of the serological tests in humans and/or dogs would have had an impact on the estimates of the proportionality coefficients $\beta_{b}$ and $\beta_{h}$, particularly if the sensitivity ratio dog/man (for $\beta_{h}$) or dog/cattle (for $\beta_{b}$) departed markedly from 1. Nevertheless, it would not have affected the model selection procedure.

Given that RVFV infection confers long-lasting immunity of more than 20 years in humans, and is considered lifelong in ruminants [38,55], we assumed that all RVFV-exposed individuals that were infected (including dogs) would remain positive for life without seroreversion, or that at least the RVFV antibody response would not wane until the age at sampling (maximum 12 years). From the 9 tested serocatalytic models, the one that provided the best fit assumed that RVFV FOIs in human and in cattle were both proportional to the RVFV FOI in dogs. Estimated RVFV FOIs in these three species showed very little variation before 2021, suggesting that the virus circulated at a low level during these years (Figure 2). These findings corroborate previous results, showing a very low-level transmission during inter-epidemic/inter-epizootic periods in Madagascar [30,45,56,57]. Olive et al. have shown that RVFV FOI in Malagasy ruminants varied over time but also by ecological regions [58]. As all municipalities in our study were from the same district (Ifanadiana), we assumed that there was no spatial variation in RVFV FOIs at the district scale. Although the abundance and diversity of mosquitoes may vary from the rainforest to rainforest edge and savanna biotope, which could result in significant variation in RVFV vectorial transmission [59], there was no consistent spatial trend in RVFV seroprevalence across host species in our study (Figure 2).

We chose to build models with a time-varying FOI to control for the yearly variations in RVFV FOIs. Given the limited number of samples from cattle and dogs born between 2011 and 2016, and to address the potential bias of precision in the year of birth of these older animals, we assumed that the RVFV FOIs through these years were constant. Keeping these data allowed us to have more statistical power and precision in parameter estimations. Despite this assumption, our estimate of RVFV FOIs exerted on cattle from 2011 to 2016 (0.009; IC 95%: 2 × 10^−4^–0.035) was within the median range of inter-epizootic RVFV FOIs estimated values from mid-2010 to mid-2014 (0.009 to 0.033) in the eastern eco-regions of Madagascar [58].

While sentinel animals were commonly used as part of an early warning system when animals are infected before humans and monitored periodically, they can also be used in epidemiological surveillance using retrospective data to evaluate the circulation of pathogens, describe the incidence and prevalence of infections over time, verify the persistence of pathogen circulation after an outbreak and test hypotheses about the ecology and epidemiology of pathogens [4,60]. Here, we found a proportional relationship between the RVFV FOI exerted on dogs and that exerted on humans and cattle to evaluate the circulation of RVFV. Further studies in other ecological regions are needed to confirm the existence of this proportional relationship, and to document the variations in the proportionality parameters, according to the vector diversity in these regions, their relative abundance, and trophic preferences, but also according to dogs’ lifestyles.

## 5. Conclusions

Besides RVFV, the circulation of several arboviruses, including West Nile virus, Usutu virus, dengue virus, and chikungunya virus, has been documented in Madagascar [61,62], and the use of dogs as sentinel animals has been suggested for *Yersinia pestis* in Madagascar [6,63]. The proportional relationship between the RVFV FOI exerted on dogs and that exerted on humans and cattle suggested that sampling dogs could allow us to identify and prioritize risk areas for RVFV, especially when human serological data are not available, and access to ruminants is limited for logistical reasons. These results pave the way for an innovative surveillance method for RVFV in Madagascar and other RVF infected countries, but also for zoonotic pathogens.

## Figures and Tables

**Table 1:** Number of individuals tested and serological results by year of birth and by species.

	DOGS			CATTLE			HUMANS		
Birth Year	Tested	Positive	% Positive [95% CI]	Tested	Positive	% Positive [95% CI]	Tested	Positive	% Positive [95% CI]
2011– 2016	64	3	4.69 [0.1–13.1]	11	4	36.4 [10.1–69.2]	305	56	18.4 [14.2–23.2]
2017	18	0	0[0–18.6]	9	1	11.1 [0.3–48.3]	56	10	17.9 [8.9–30.4]
2018	26	0	0[0–13.2]	13	5	38.46 [13.9–68.4]	42	7	16.7 [6.9–31.4]
2019	31	5	16.1 [5.4–33.7]	17	6	35.3 [14.2–61.7]	37	5	13.5 [4.5–28.8]
2020	61	5	8.2 [2.7–18.1]	11	4	36.4 [10.9–69.2]	38	5	13.2 [4.4–28.1]
2021	58	6	10.3 [3.9–21.2]	23	8	34.8 [16.4–57.3]	8	3	37.5 [8.5–75.5]
2022	139	4	2.88 [0.8–7.2]	19	3	15.8 [3.4–39.6]	0	0	-
2023	116	0	0[0–3.1]	32	2	6.3 [0.8–20.8]	0	0	-
TOTAL	513	23	4.5 [2.9–6.7]	135	33	24.4 [17.5–32.6]	486	86	17.7 [14.4–21.9]

**Table 2:** Description of serocatalytic models, with corresponding number of estimated parameters and deviance information criterion values.

Model	Description of the Relationship of RVFV FOI in Dogs to		Number of Estimated Parameters	DIC
	RVFV FOI in Humans	RVFV FOI in Cattle		
Model 0	H0	H0	23	810.7
Model 1	H1	H0	18	801.8
Model 2	H2	H0	26	819.9
Model 3	H0	H1	16	798.9
Model 4	H1	H1	11	790.3
Model 5	H2	H1	19	809.4
Model 6	H0	H2	24	818.2
Model 7	H1	H2	19	813.8
Model 8	H2	H2	19	809.2

DIC: Deviance Information Criterion; FOI: Force of Infection; H0: null hypothesis; H1: first alternative hypothesis; H2: second alternative hypothesis.

## Data Availability

Data analyzed during this study is presented within the paper and its Supplementary Materials.

## Supplementary Materials

The following supporting information can be downloaded at https://www.mdpi.com/article/10.3390/v17111461/s1, Figure S1: An example of autocorrelation graph from autocorrelation function (ACF), model 4. (PDF); Figure S2: Distribution of individual S/N% values (dogs and cattle) and MFI values (Humans) from cELISA and MIA serological analyses, respectively; Table S1: Dog, human and cattle database with age and year of exposure to RVFV. (XLSX); Table S2: Effective sample size (NEEF), NEEF ratio, Rhat, and values of estimated parameters per model. Estimated parameter values, NEEF and Rhat values are presented. Details of parameters: FOI is the force of infection, B is bovine (cattle), C is canine (dogs), 16 is the 2011 to 2016 years, 17 to 24 are the years 2017 to 2024, CC is the contact fraction of RVFV FOI in dogs, CV is the vectorial fraction of RVFV FOI in dogs. (XLSX); Table S3: Individuals tested and positive for anti-RVFV antibodies by communes and species, with Chi-2/Fisher exact *p*-values. In dogs and humans, RVFV seroprevalence in Tsaratanana was the highest, with 10.9% (7/64, 95% CI: [4.5–21.2]) and 29.5% (43/146, 95% CI: [22.2–37.6]), respectively. However, without considering Tsaratanana, no spatial difference in RVFV seroprevalence was found between localities in the three species. (XLSX).

## Abbreviations

The following abbreviations are used in this manuscript:

ACF	Autocorrelation Function
cELISA	competitive Enzyme-Linked Immunosorbent Assay
CI	Confidence intervals
CrI	Credible Intervals
DBS	Dry Blood Spot
DENV	Dengue Virus
DIC	Deviance Information Criterion
FOI	Force Of Infection
H0	null hypothesis
H1	first alternative hypothesis
H2	second alternative hypothesis
IHOPE	Ifanadiana Health Outcomes and Prosperity longitudinal Evaluation
IgG	Immunoglobulins of type G
INSTAT	Institut National de la Statistique
IPM	Institut Pasteur de Madagascar
JEV	Japanese Encephalitis Virus
MIA	microsphere-based immunoassay
MCMC	Markov Chain Monte Carlo
NEFF	Effective Sample Size
NP	Nucleoprotein
RVF	Rift Valley Fever
RVFV	Rift Valley Fever Virus
WNV	West Nile Virus

## References

[1] World Bank (2024). World Bank Navigating Two Decades of High Poverty and Charting a Course for Change in Madagascar: Poverty and Equity Assessment, February 2024.

[2] Bonds M.H., Ouenzar M.A., Garchitorena A., Cordier L.F., McCarty M.G., Rich M.L., Andriamihaja B., Haruna J., Farmer P.E. (2018). Madagascar Can Build Stronger Health Systems to Fight Plague and Prevent the next Epidemic. PLoS Negl. Trop. Dis..

[3] Neo J.P.S., Tan B.H. (2017). The Use of Animals as a Surveillance Tool for Monitoring Environmental Health Hazards, Human Health Hazards and Bioterrorism. Vet. Microbiol..

[4] Halliday J.E.B., Meredith A.L., Knobel D.L., Shaw D.J., Bronsvoort B.M.d.C., Cleaveland S. (2007). A Framework for Evaluating Animals as Sentinels for Infectious Disease Surveillance. J. R. Soc. Interface.

[5] Resnick M.P., Grunenwald P., Blackmar D., Hailey C., Bueno R., Murray K.O. (2008). Juvenile Dogs as Potential Sentinels for West Nile Virus Surveillance. Zoonoses Public Health.

[6] Bowser N.H., Anderson N.E. (2018). Dogs (*Canis familiaris*) as Sentinels for Human Infectious Disease and Application to Canadian Populations: A Systematic Review. Vet. Sci..

[7] Davoust B., Leparc-Goffart I., Demoncheaux J.-P., Tine R., Diarra M., Trombini G., Mediannikov O., Marié J.-L. (2014). Serologic Surveillance for West Nile Virus in Dogs, Africa. Emerg. Infect. Dis..

[8] Durand B., Haskouri H., Lowenski S., Vachiery N., Beck C., Lecollinet S. (2016). Seroprevalence of West Nile and Usutu Viruses in Military Working Horses and Dogs, Morocco, 2012: Dog as an Alternative WNV Sentinel Species?. Epidemiol. Infect..

[9] Thongyuan S., Kittayapong P. (2017). First Evidence of Dengue Infection in Domestic Dogs Living in Different Ecological Settings in Thailand. PLoS ONE.

[10] Shimoda H., Ohno Y., Mochizuki M., Iwata H., Okuda M., Maeda K. (2010). Dogs as Sentinels for Human Infection with Japanese Encephalitis Virus. Emerg. Infect. Dis..

[11] Adams M.J., Lefkowitz E.J., King A.M.Q., Harrach B., Harrison R.L., Knowles N.J., Kropinski A.M., Krupovic M., Kuhn J.H., Mushegian A.R. (2017). Changes to Taxonomy and the International Code of Virus Classification and Nomenclature Ratified by the International Committee on Taxonomy of Viruses (2017). Arch. Virol..

[12] Daubney R., Hudson J.R., Garnham P.C. (1931). Enzootic Hepatitis or Rift Valley Fever. An Undescribed Virus Disease of Sheep Cattle and Man from East Africa. J. Pathol. Bacteriol..

[13] Budasha N., Gonzalez J., Sebhatu T., Ezama A. (2018). Rift Valley Fever Seroprevalence and Abortion Frequency among Livestock of Kisoro District, South Western Uganda (2016): A Prerequisite for Zoonotic Infection. BMC Vet. Res..

[14] Anywaine Z., Lule S.A., Hansen C., Warimwe G., Elliott A. (2022). Clinical Manifestations of Rift Valley Fever in Humans: Systematic Review and Meta-Analysis. PLoS Negl. Trop. Dis..

[15] Fontenille D. (1989). Etude Des Circuits de Vection d’arbovirus, à Madagascar.

[16] Morvan J., Saluzzo J.F., Fontenille D., Rollin P., Coulangés P. (1991). Rift valley fever on the east coast of Madagascar. Res. Virol..

[17] Morvan J., Rollin P., Laventure S., Rakotoarivony I., Guillot J. (1992). Rift Valley fever epizootic in the central highlands of Madagascar. Res. Virol..

[18] Andriamandimby S.F., Randrianarivo-Solofoniaina A.E., Jeanmaire E.M., Ravololomanana L., Razafimanantsoa L.T., Rakotojoelinandrasana T., Razainirina J., Hoffmann J., Ravalohery J.-P., Rafisandratantsoa J.-T. (2010). Rift Valley Fever during Rainy Seasons, Madagascar, 2008 and 2009. Emerg. Infect. Dis..

[19] Harimanana A., Andriamandimby S., Ranoaritiana D., Randrianasolo L., Irinantenaina J., Ranoelison N., Rafisandrantatsoa J., Ankasitrahana M., Raherinandrasana A., Andriamahatana M. (2024). The Re-Emergence of Rift Valley Fever in Mananjary District, Madagascar in 2021: A Call for Action. Pathogens.

[20] Ratsitorahina M., Rasambainarivo J.H., Raharimanana S., Rakotonandrasana H., Andriamiarisoa M.-P., Rakalomanana F.A., Richard V. (2009). Dog Ecology and Demography in Antananarivo, 2007. BMC Vet. Res..

[21] Kshirsagar A.R., Applebaum J.W., Randriana Z., Rajaonarivelo T., Rafaliarison R.R., Farris Z.J., Valenta K. (2020). Human-Dog Relationships across Communities Surrounding Ranomafana and Andasibe-Mantadia National Parks, Madagascar. J. Ethnobiol..

[22] Cleaveland S., Meslin F.X., Breiman R. (2006). Dogs Can Play Useful Role as Sentinel Hosts for Disease. Nature.

[23] Becquart P., Bohou Kombila L., Mebaley T.N., Paupy C., Garcia D., Nesi N., Olive M.-M., Vanhomwegen J., Boundenga L., Mombo I.M. (2024). Evidence for Circulation of Rift Valley Fever Virus in Wildlife and Domestic Animals in a Forest Environment in Gabon, Central Africa. PLoS Negl. Trop. Dis..

[24] Hoogstraal H., Meegan J.M., Khalil G.M., Adham F.K. (1979). The Rift Valley Fever Epizootic in Egypt 1977–1978. 2. Ecological and Entomological Studies. Trans. R. Soc. Trop. Med. Hyg..

[25] Ihantamalala F.A., Bonds M.H., Randriamihaja M., Rakotonirina L., Herbreteau V., Révillion C., Rakotoarimanana S., Cowley G., Andriatiana T.A., Mayfield A. (2021). Geographic Barriers to Establishing a Successful Hospital Referral System in Rural Madagascar. BMJ Glob. Health.

[26] Evans M.V., Bonds M.H., Cordier L.F., Drake J.M., Ihantamalala F., Haruna J., Miller A.C., Murdock C.C., Randriamanambtsoa M., Raza-Fanomezanjanahary E.M. (2021). Socio-Demographic, Not Environmental, Risk Factors Explain Fine-Scale Spatial Patterns of Diarrhoeal Disease in Ifanadiana, Rural Madagascar. Proc. R. Soc. B Biol. Sci..

[27] CREAM (2013). Monographie de la Région Vatovavy Fitovinany.

[28] Cordier L.F., Kalaris K., Rakotonanahary R.J.L., Rakotonirina L., Haruna J., Mayfield A., Marovavy L., McCarty M.G., Aina A.T., Ratsimbazafy B. (2020). Networks of Care in Rural Madagascar for Achieving Universal Health Coverage in Ifanadiana District. Health Syst. Reform.

[29] Miller A.C., Garchitorena A., Rabeza V., Randriamanambintsoa M., Rahaniraka Razanadrakato H.-T., Cordier L., Ouenzar M.A., Murray M.B., Thomson D.R., Bonds M.H. (2018). Cohort Profile: Ifanadiana Health Outcomes and Prosperity Longitudinal Evaluation (IHOPE). Int. J. Epidemiol..

[30] Olive M.-M., Chevalier V., Grosbois V., Tran A., Andriamandimby S.-F., Durand B., Ravalohery J.-P., Andriamamonjy S., Rakotomanana F., Rogier C. (2016). Integrated Analysis of Environment, Cattle and Human Serological Data: Risks and Mechanisms of Transmission of Rift Valley Fever in Madagascar. PLoS Negl. Trop. Dis..

[31] Miller A.C., Ramananjato R.H., Garchitorena A., Rabeza V.R., Gikic D., Cripps A., Cordier L., Razanadrakato H.-T.R., Randriamanambintsoa M., Hall L. (2017). Baseline Population Health Conditions Ahead of a Health System Strengthening Program in Rural Madagascar. Glob. Health Action.

[32] Kielkopf C.L., Bauer W., Urbatsch I.L. (2020). Bradford Assay for Determining Protein Concentration. Cold Spring Harb. Protoc..

[33] Melnykov V., Maitra R. (2010). Finite Mixture Models and Model-Based Clustering. Stat. Surv..

[34] Pérez-Ramírez E., Cano-Gómez C., Llorente F., Adžić B., Al Ameer M., Djadjovski I., Hage J., Mellouli F., Goletić T., Hovsepyan H. (2020). External Quality Assessment of Rift Valley Fever Diagnosis in 17 Veterinary Laboratories of the Mediterranean and Black Sea Regions. PLoS ONE.

[35] Pédarrieu A., Mellouli F., Khallouki H., Zro K., Sebbar G., Sghaier S., Madani H., Bouayed N., Lô M., Diop M. (2021). External Quality Assessment of Rift Valley Fever Diagnosis in Countries at Risk of the Disease: African, Indian Ocean and Middle-East Regions. PLoS ONE.

[36] Berguido F.J., Settypalli T.B.K., Mbuyi C.G.T., Bakhom M.T., van Vuren P.J., Pawęska J.T., Cattoli G., Grabherr R., Lamien C.E. (2024). Development of a Luminex-Based Assay for the Detection of Anti-Capripoxvirus and Rift Valley Fever Virus Antibodies in Domestic Ruminants. Virol. J..

[37] Hens N., Aerts M., Faes C., Shkedy Z., Lejeune O., Damme P.V., Beutels P. (2010). Seventy-Five Years of Estimating the Force of Infection from Current Status Data. Epidemiol. Infect..

[38] Brown R.D., Scott G.R., Dalling T. (1957). Persistence of Antibodies to Rift Valley Fever in Man. Lancet.

[39] Matiko M.K., Salekwa L.P., Kasanga C.J., Kimera S.I., Evander M., Nyangi W.P. (2018). Serological Evidence of Inter-Epizootic/Inter-Epidemic Circulation of Rift Valley Fever Virus in Domestic Cattle in Kyela and Morogoro, Tanzania. PLoS Negl. Trop. Dis..

[40] Wright D., Kortekaas J., Bowden T.A., Warimwe G.M. (2019). Rift Valley Fever: Biology and Epidemiology. J. Gen. Virol..

[41] Wright D., Allen E.R., Clark M.H.A., Gitonga J.N., Karanja H.K., Hulswit R.J.G., Taylor I., Biswas S., Marshall J., Mwololo D. (2020). Naturally Acquired Rift Valley Fever Virus Neutralizing Antibodies Predominantly Target the Gn Glycoprotein. iScience.

[42] Hay J.A., Routledge I., Takahashi S. (2024). Serodynamics: A Primer and Synthetic Review of Methods for Epidemiological Inference Using Serological Data. Epidemics.

[43] Cucunubá Z.M., Nouvellet P., Conteh L., Vera M.J., Angulo V.M., Dib J.C., Parra-Henao G.J., Basáñez M.G. (2017). Modelling Historical Changes in the Force-of-Infection of Chagas Disease to Inform Control and Elimination Programmes: Application in Colombia. BMJ Glob. Health.

[44] Jeanmaire E., Rabenarivahiny R., Biarmann M., Rabibisoa L., Ravaomanana F., Randriamparany T., Andriamandimby S., Diaw C., Fenozara P., De La Rocque S. (2011). Prevalence of Rift Valley Fever Infection in Ruminants in Madagascar After the 2008 Outbreak. Vector-Borne Zoonotic Dis..

[45] Gray G., Anderson B., LaBeaud A., Héraud J., Fèvre E., Andriamandimby S., Cook E., Dahir S., de Glanville W., Heil G. (2015). Seroepidemiological Study of Interepidemic Rift Valley Fever Virus Infection Among Persons with Intense Ruminant Exposure in Madagascar and Kenya. Am. J. Trop. Med. Hyg..

[46] Clark M., Warimwe G., Di Nardo A., Lyons N., Gubbins S. (2018). Systematic Literature Review of Rift Valley Fever Virus Seroprevalence in Livestock, Wildlife and Humans in Africa from 1968 to 2016. PLoS Negl. Trop. Dis..

[47] Ahmed Kamal S. (2011). Observations on Rift Valley Fever Virus and Vaccines in Egypt. Virol. J..

[48] Hartman A. (2017). Rift Valley Fever. Clin. Lab. Med..

[49] Chevalier V., Pépin M., Plée L., Lancelot R. (2010). Rift Valley Fever—A Threat for Europe?. Eurosurveillance.

[50] Richards S.L., Ponnusamy L., Unnasch T.R., Hassan H.K., Apperson C.S. (2006). Host-Feeding Patterns of *Aedes albopictus* (Diptera: Culicidae) in Relation to Availability of Human and Domestic Animals in Suburban Landscapes of Central North Carolina. J. Med. Entomol..

[51] Ponlawat A., Harrington L.C. (2005). Blood Feeding Patterns of *Aedes aegypti* and *Aedes albopictus* in Thailand. J. Med. Entomol..

[52] Garcia-Rejon J.E., Blitvich B.J., Farfan-Ale J.A., Loroño-Pino M.A., Chi Chim W.A., Flores-Flores L.F., Rosado-Paredes E., Baak-Baak C., Perez-Mutul J., Suarez-Solis V. (2010). Host-Feeding Preference of the Mosquito, Culex Quinquefasciatus, in Yucatan State, Mexico. J. Insect Sci..

[53] Kortekaas J., Kant J., Vloet R., Cêtre-Sossah C., Marianneau P., Lacôte S., Banyard A., Jeffries C., Eiden M., Groschup M. (2012). European Ring Trial to Evaluate ELISAs for the Diagnosis of Infection with Rift Valley Fever Virus. J. Virol. Methods.

[54] Surtees R., Stern D., Ahrens K., Kromarek N., Lander A., Kreher P., Weiss S., Hewson R., Punch E.K., Barr J.N. (2020). Development of a Multiplex Microsphere Immunoassay for the Detection of Antibodies against Highly Pathogenic Viruses in Human and Animal Serum Samples. PLoS Negl. Trop. Dis..

[55] Geering W.A., Davies F.G., Martin V. (2002). Preparation of Rift Valley Fever Contingency Plans.

[56] Chevalier V., Rakotondrafara T., Jourdan M., Héraud J., Andriamanivo H., Durand B., Ravaomanana J., Rollin P., Rakotondravao R. (2011). An Unexpected Recurrent Transmission of Rift Valley Fever Virus in Cattle in a Temperate and Mountainous Area of Madagascar. PLoS Negl. Trop. Dis..

[57] Nicolas G., Durand B., Rakotoarimanana T., Lacôte S., Chevalier V., Marianneau P. (2013). A 3-Year Serological and Virological Cattle Follow-Up in Madagascar Highlands Suggests a Non-Classical Transmission Route of Rift Valley Fever Virus. Am. J. Trop. Med. Hyg..

[58] Olive M.-M., Grosbois V., Tran A., Nomenjanahary L.A., Rakotoarinoro M., Andriamandimby S.-F., Rogier C., Heraud J.-M., Chevalier V. (2017). Reconstruction of Rift Valley Fever Transmission Dynamics in Madagascar: Estimation of Force of Infection from Seroprevalence Surveys Using Bayesian Modelling. Sci. Rep..

[59] Tantely L., Rakotoniaina J.-C., Tata E., Andrianaivolambo L., Razafindrasata F., Fontenille D., Élissa N. (2013). Biology of Mosquitoes That Are Potential Vectors of Rift Valley Fever Virus in Different Biotopes of the Central Highlands of Madagascar. J. Med. Entomol..

[60] Toma B. (2009). La fonction sentinelle en epidemiologie. Epidémiol. et santé anim..

[61] Broban A., Olive M.-M., Tantely M.L., Dorsemans A.-C., Rakotomanana F., Ravalohery J.-P., Rogier C., Heraud J.-M., Andriamandimby S.F. (2023). Seroprevalence of IgG Antibodies Directed against Dengue, Chikungunya and West Nile Viruses and Associated Risk Factors in Madagascar, 2011 to 2013. Viruses.

[62] Chevalier V., Marsot M., Molia S., Rasamoelina H., Rakotondravao R., Pedrono M., Lowenski S., Durand B., Lecollinet S., Beck C. (2020). Serological Evidence of West Nile and Usutu Viruses Circulation in Domestic and Wild Birds in Wetlands of Mali and Madagascar in 2008. Int. J. Environ. Res. Public. Health.

[63] Rajerison M., Andrianaivoarimanana V., Ratsitorahina M., Rahelinirina S., Chanteau S., Telfer S., Rahalison L. (2021). Field Assessment of Dog as Sentinel Animal for Plague in Endemic Foci of Madagascar. Integr. Zool..

