# Supplementary figures and images for: A Multi-Host Approach to Quantitatively Assess the Role of Dogs as Sentinels for Rift Valley Fever Virus (RVFV) Surveillance in Madagascar
Source: Viruses. 2025 Oct 31;17(11):1461. doi: 10.3390/v17111461 (PMC12656841; doi:10.3390/v17111461)

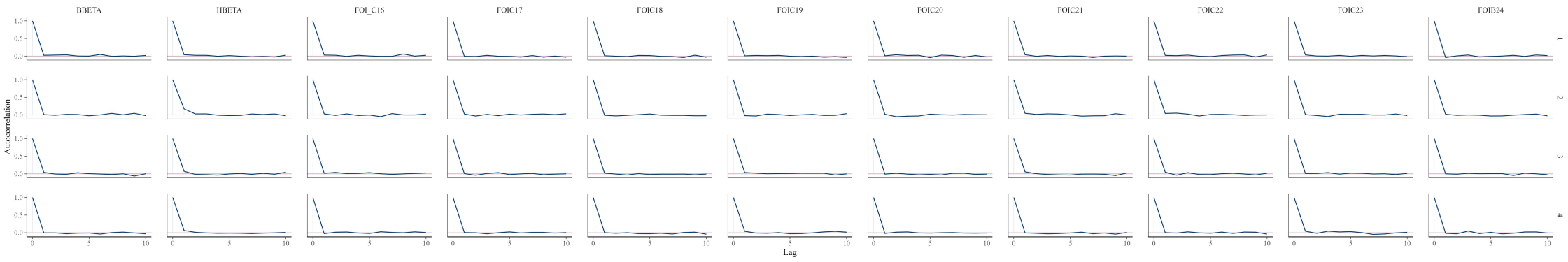

Supplement: Supplementary file 1 [file viruses-17-01461-s001.zip › Figure_S1.pdf]

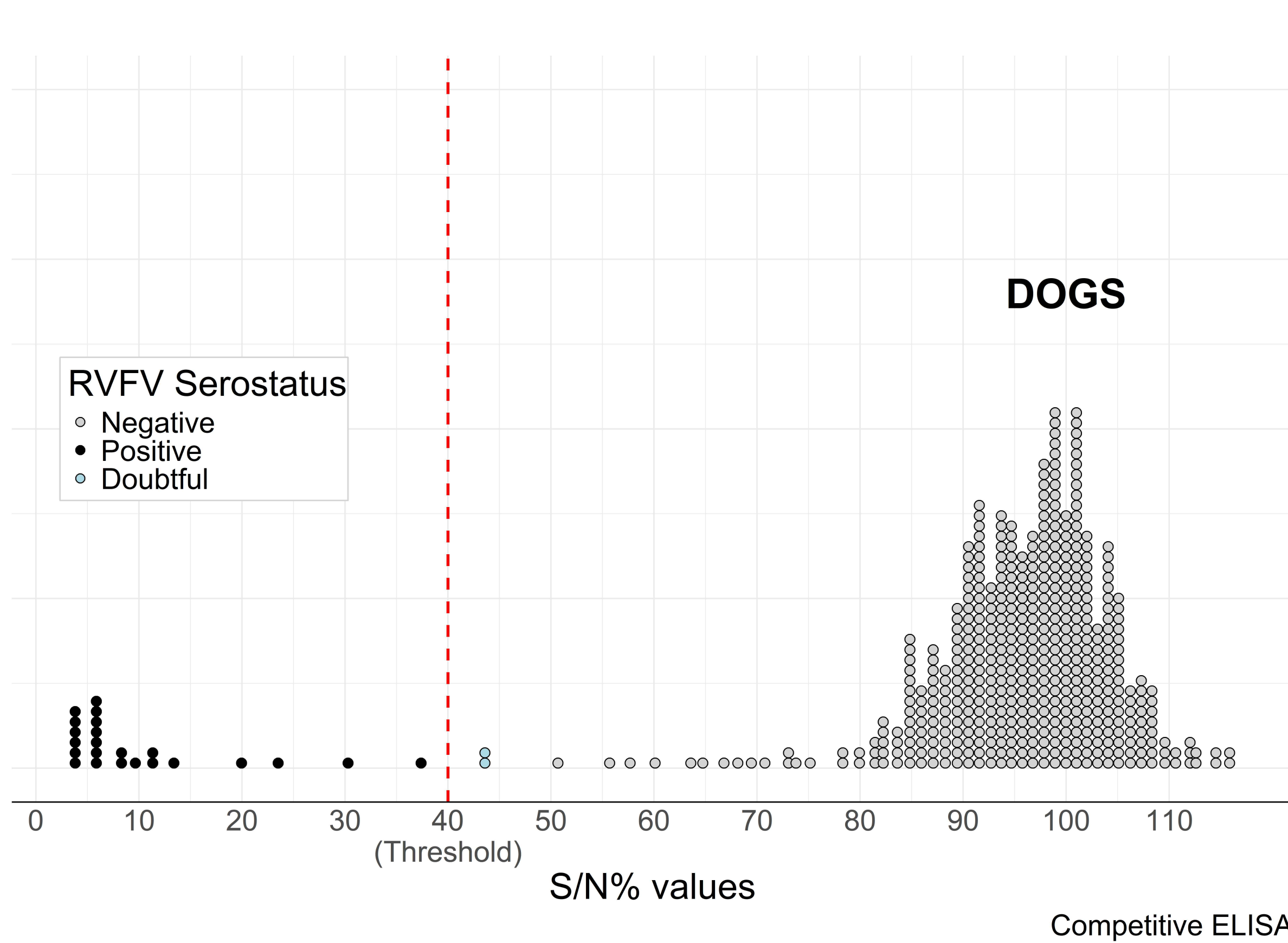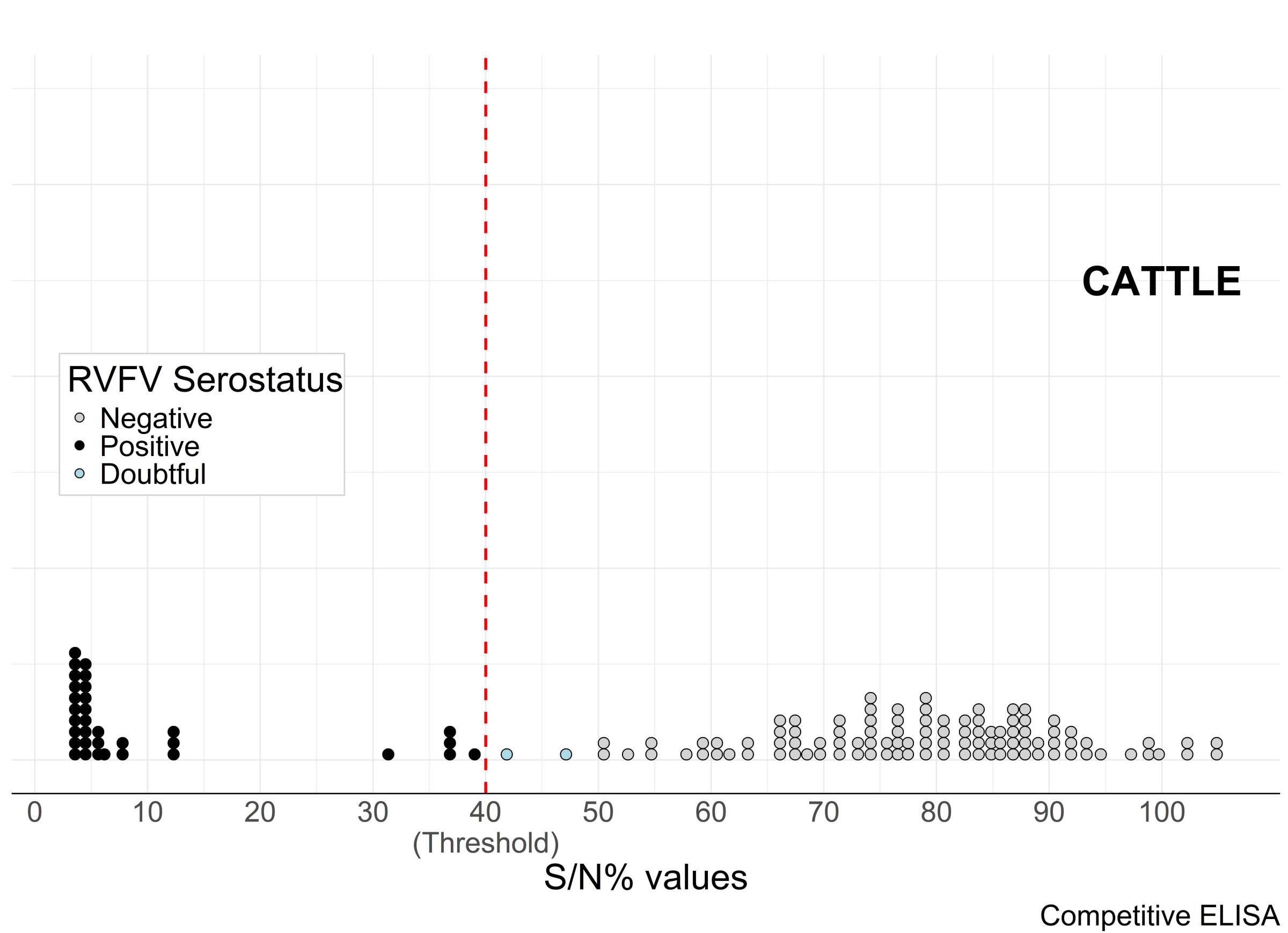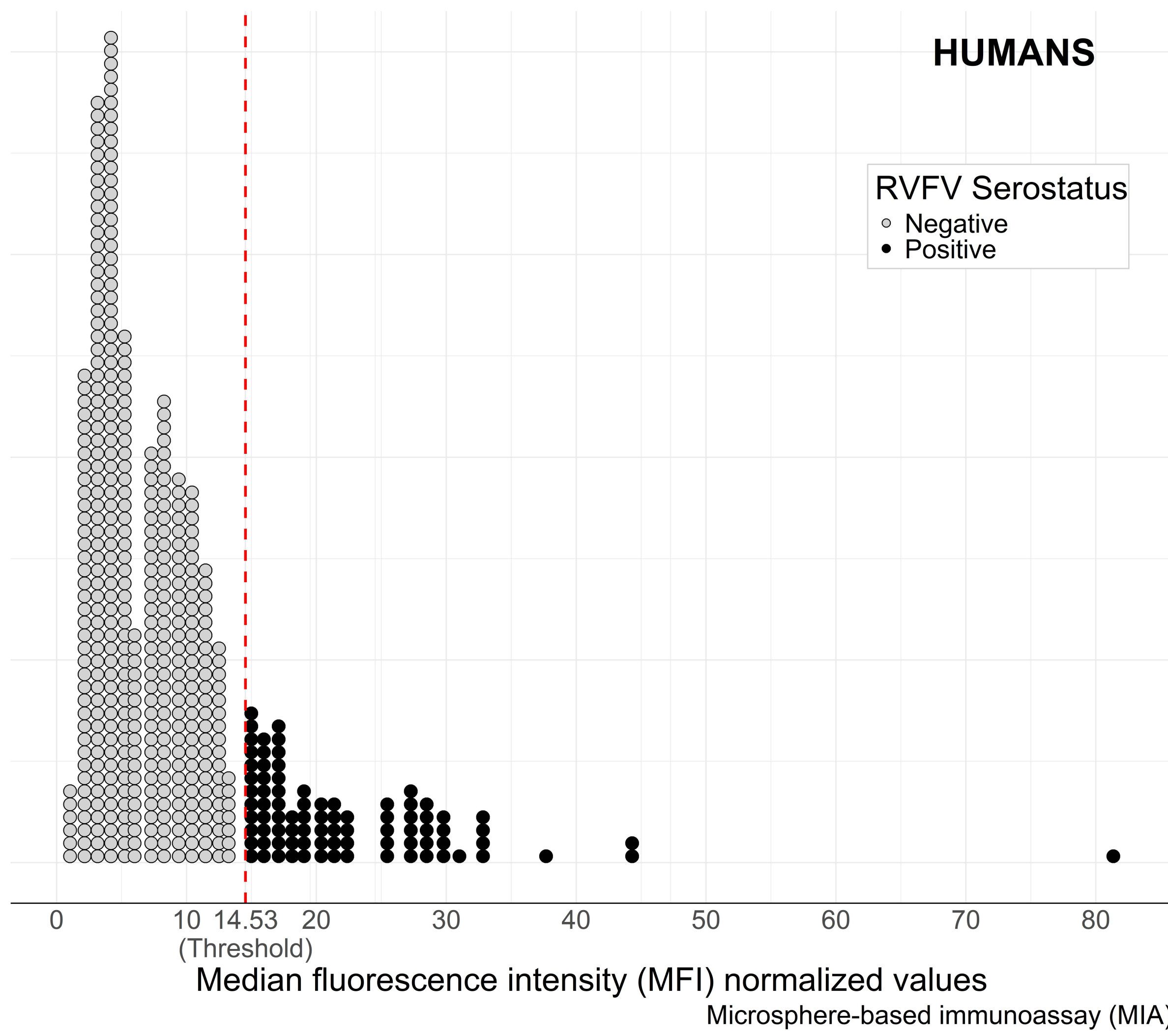

Supplement: Supplementary file 1 [file viruses-17-01461-s001.zip › Figure_S2.pdf]
